# Radiomics-Based Muscle Quality Assessment from Multi-Site Multi-Vendor MRI

**DOI:** 10.21203/rs.3.rs-10845667/v1

**Published:** 2026-10-07

**Authors:** Ozkan Cigdem, Eros Montin, Salim Bin Ghouth, Akshay Chaudhari, Donnie Cameron, Christopher M. Bergeron, Jan Bergeron, Luigi Ferrucci, Garry E. Gold, Riccardo Lattanzi, Cem M. Deniz, Valentina Mazzoli

**Affiliations:** 1 Department of Radiology, New York University Grossman School of Medicine, New York, United States.; 2 Department of Radiology, Stanford University, Stanford, United States.; 3 Department of Medical Imaging, Radboudumc, Nijmegen, the Netherlands.; 4 Intramural Research Program, National Institute on Aging, National Institutes of Health, Maryland, United States.

**Keywords:** Sarcopenia, Dixon MRI, thigh muscle, radiomics, texture analysis

## Abstract

Muscle strength is critical for mobility, and its decline with aging contributes to frailty, falls, and loss of independence. Muscle cross-sectional area (CSA), which can be measured on MRI, only reflects muscle quantity and may not fully capture age-related changes in muscle composition, which significantly contribute to loss of muscle strength in older adults. We investigated strength prediction using multi-center/multi-scanner Dixon MRI from 141 adults across three cohorts: 82 healthy adults (Philips, 22–94 years), 24 healthy adults (GE, 30–79 years), and 35 older adults (Siemens, 70–91 years), including healthy, pre-frail, and sarcopenic participants. CSA was a strong predictor of muscle strength in the healthy cohorts, but radiomics features provided additional value when older adults were included. Fat-fraction radiomics showed the strongest and most consistent performance across cohort combinations, with the best results in the full combined cohort (R^2^=0.64 versus 0.42 for CSA alone). Water-only radiomics also retained predictive value, although generally less strongly than fat-fraction features. The most informative radiomics features reflected muscle size, signal magnitude, and internal heterogeneity, and SHAP analysis linked higher intramuscular heterogeneity to reduced strength. Taken together, these findings suggest that radiomics can capture muscle quality features that affect muscle force-generating capacity beyond size alone, particularly in older adults, and support automated MRI-based assessment of muscle composition and function, which potentially help future sarcopenia screening and functional risk stratification.

## 1 Introduction

Muscle strength is a fundamental biomarker of neuromuscular health and plays a central role in maintaining mobility, functional independence, and overall physiological well-being [1, 2]. Sarcopenia, an age-related syndrome characterized by a progressive loss of muscle mass and strength, is a major contributor to mobility decline, falls, fractures, and early mortality in older adults [3–6]. As the global population continues to age, the healthcare burden associated with sarcopenia is expected to rise substantially [7], underscoring the urgent need for early identification and effective management strategies.

Sarcopenia is a complex condition, with many manifestations including deposition of fat and fibrosis in muscle, inflammation, and muscle fiber atrophy. These early changes likely precede global loss of muscle mass and decreased physical function, and collectively contribute to lower muscle strength by reducing the amount of contractile tissue [4]. Therefore, methods capable of detecting early and potentially reversible changes in muscle composition may be particularly valuable for identifying functional decline before visible atrophy becomes evident.

Muscle mass, a major determinant of force production and function, can be determined using several imaging techniques, including computed tomography, dual-energy X-ray absorptiometry (DXA), and ultrasound [8, 9]. However, these provide limited insight into early and reversible changes in muscle composition [10, 11]. In contrast, magnetic resonance imaging (MRI) provides not only three-dimensional assessments of muscle size and morphology, but can also identify early compositional changes [12, 13]. In particular, chemical-shift-based water-fat-separation, or ’Dixon’, MRI [14] allows precise quantification of intramuscular fat and facilitates reliable muscle segmentation. Recent studies have demonstrated that incorporating the metrics of muscle composition can improve the prediction of quadriceps strength over muscle cross-sectional area (CSA) alone [12].

Muscle strength—assessed using knee extension torque—is a key functional outcome associated with mobility, fall risk, and functional decline. However, direct measurement of knee extension torque requires specialized isokinetic dynamometry, which is not accessible in routine clinical practice. As a result, existing studies use muscle-quantity measures such as CSA or muscle volume as surrogate markers of strength, since muscle size generally correlates positively with strength [9, 15, 16]. Although informative, these metrics overlook finer-grained compositional patterns and intramuscular heterogeneity that may signal early pathological changes and contribute meaningfully to force generation. Radiomics offers a complementary framework for transforming medical images into high-dimensional quantitative descriptors that capture tissue shape, texture, intensity distributions, and spatial complexity [17]. Applied to muscle MRI, radiomics has the potential to reveal subtle patterns in muscle composition that are not detectable with coarse morphological imaging, and to provide a better assessment of the patient strength/function compared to metrics of muscle quantity such as CSA [18].

Prior studies have shown that CT-derived skeletal muscle radiomics can identify overt sarcopenia in advanced lung cancer patients [19, 20] and osteoporosis [21], while ultrasound-based texture analysis using radiomics has been associated with frailty and adverse aging-related outcomes [22, 23]. Recent CT studies in older adults showed that muscle radiomic features were associated with muscle strength, physical performance, and longitudinal functional decline beyond conventional CT-derived CSA and density [24, 25], suggesting the ability of radiomics to identify features of muscle quality which are not captured by simple morphological assessments. MRI offers quantitative assessments of muscle composition, which directly relates to muscle strength, but the potential of MRI-based radiomics for strength prediction remains underexplored.

In this study, our overarching aim was to leverage anatomical MR scans of the thigh to address a critical need in musculoskeletal imaging: the early identification of sarcopenia-related muscle changes before visible muscle atrophy becomes apparent. Because early muscle deterioration is associated with impaired muscle function, we focused on predicting quadriceps strength, measured as knee-extension torque, directly from MR exams. Specifically, we evaluated whether radiomics features extracted from Dixon images could predict quadriceps muscle strength more effectively than conventional CSA measures. We hypothesized that compositional features related to muscle function can be automatically derived from MRI using radiomics and that these features provide improved prediction of quadriceps muscle strength compared with CSA alone or with CSA combined with mean fat fraction.

## 2 Results

Table 1 summarizes strength prediction performance using radiomics features extracted from water-only images, fat-fraction images, and their combination across different dataset combinations. In the healthy cohorts (Dataset-1+Dataset-2), CSA and fat fraction radiomics performed similarly (*R*^2^ = 0.58 vs 0.59; Δ*R*^2^ = 0.008 [−0.095, 0.111]), whereas water-only radiomics performed worse than CSA. When Dataset-3 was included, radiomics generally outperformed CSA. For Dataset-1+Dataset-3, all radiomics models significantly improved prediction over CSA, with the best performance from combined water+fat fraction features (*R*^2^ = 0.58 vs 0.24; Δ*R*^2^ = 0.338 [0.146, 0.645]; ΔRMSE= 10.67 Nm [3.99, 16.37]). For Dataset-2+Dataset-3, radiomics models showed numerically higher performance than CSA, with the highest *R*^2^ for water+fat fraction (0.67 vs 0.54), although improvements were not statistically significant. In the full combined cohort, fat fraction radiomics achieved the strongest performance and significantly improved prediction over CSA (*R*^2^ = 0.64 vs 0.42; Δ*R*^2^ = 0.218 [0.085, 0.399]; ΔRMSE= 8.02 Nm [3.06, 12.55]).

### Residual Error Characteristics and Agreement Analysis

For the fat fraction-based models, both CSA and radiomics showed small mean bias relative to true strength across all dataset combinations, with Bland–Altman bias ranging from −4.46 to +3.50 Nm (Figure 1). In the full combined cohort (Dataset-1+Dataset-2+Dataset-3, *n* = 38), fat fraction radiomics showed tighter agreement than CSA, with narrower limits of agreement (LOA: −59.5 to +59.3 Nm vs −76.5 to +74.1 Nm), corresponding to a reduction in LOA width from 150.5 to 118.7 Nm, together with lower MAE (23.4 vs 29.4 Nm). A similar pattern was observed for Dataset-1+Dataset-3 (*n* = 28), where fat fraction radiomics reduced agreement width from 166.0 to 125.6 Nm and lowered MAE from 33.4 to 24.8 Nm. In Dataset-2+Dataset-3 (*n* = 22), radiomics also showed narrower LOA (112.0 vs 130.8 Nm) and lower MAE (23.3 vs 26.9 Nm), although bootstrap comparisons were not statistically significant. In contrast, for Dataset-1+Dataset-2 (*n* = 24), agreement was similar between models, with only small, non-significant improvements for radiomics. Residual analysis was consistent with these findings: in dataset combinations including Dataset-3, fat fraction radiomics reduced residual spread, with lower residual standard deviation (e.g., 42.4 vs 32.0 Nm for Dataset-1+Dataset-3 and 38.4 vs 30.3 Nm for the full cohort) and lower mean absolute residuals (33.4 vs 24.8 Nm and 29.4 vs 23.4 Nm, respectively), with significant improvement in absolute error in Dataset-1+Dataset-3 and the full combined cohort. Supplementary Document Figures S.5–S.6 provide Bland–Altman plots for the water-only and combined water+fat-fraction radiomics models across all dataset combinations, while Supplementary Document S.3 (Figures S.7–S.9) provide absolute error comparisons across all dataset combinations for all radiomics models (fat-fraction, water-only, and combined water+fat-fraction).

### Selected Radiomics Features

Across the different dataset combinations, the selected radiomics features followed a biologically coherent pattern in which larger muscle size and stronger water-only signal were associated with higher predicted torque, whereas greater fat-related signal and increased textural heterogeneity were generally associated with lower predicted torque. For the Dataset-1 + Dataset-2 combination, the selected features were likewise dominated by negative heterogeneity-related effects. In the water-only models, *”local binary pattern (LBP) 3D gray level size zone matrix (GLSZM) size-zone non-uniformity”* showed a negative SHAP direction, while in the fat-fraction models, *”square root GLSZM gray-level non-uniformity”* and *”LBP 3D gray level co-occurrence matrix (GLCM) Informational Measure of Correlation (IMC)”* were also negative. These findings indicate that increasingly heterogeneous and disorganized muscle composition was linked to lower predicted torque in this cross-site setting. In contrast, the shape feature *”original shape minor axis length”* showed a positive direction, consistent with the expected contribution of larger muscle size to strength.

In the Dataset-1 + Dataset-3 analysis, the selected water-only features *”logarithm gray level dependence matrix (GLDM) gray-level non-uniformity”* and *”wavelet-high-high-high GLCM inverse variance”* both showed negative SHAP directions. Similarly, the fat-fraction feature *”LBP 3D GLCM IMC”* was negative. Overall, these results suggest that, in this dataset pairing, greater non-uniformity and altered local texture relationships were linked to reduced torque.

For the Dataset-2 + Dataset-3 combination, *”original shape maximum 2D diameter slice”* was repeatedly selected with a positive SHAP direction in both the fat-fraction and combined water-fat models, reinforcing the importance of muscle size as a predictor of torque. By contrast, fat-related texture and intensity features, including *”LBP 2D first-order total energy”*, and *”LBP 3D GLCM IMC”*, showed negative SHAP directions. The water-only feature *”wavelet-low-high-low GLDM dependence entropy”* was also negative, again suggesting that greater textural irregularity and compositional disruption were associated with lower strength.

In the combined analysis of all three datasets, the water-only feature *”first-order energy”* showed a positive SHAP direction, indicating that higher muscle signal energy contributed to greater predicted torque. In contrast, several heterogeneity-related features demonstrated negative SHAP directions, including *”wavelet-low-high-low GLDM dependence entropy”*, *”gray-level non-uniformity”*, and *”size-zone non-uniformity”*. These features reflect greater disorder, non-uniform gray-level distribution, and irregular zone structure within the muscle, all of which are consistent with lower strength. Fat-fraction features selected in all models also showed predominantly negative directions, including *”LBP 2D first-order total energy”* and *”LBP 3D GLCM IMC”*, suggesting that greater fat-related signal and more complex fat-texture patterns were associated with reduced torque.

Taken together, the most consistent positive contributors across models were shape-related measures and water-only energy features, particularly *”maximum 2D diameter slice”*, *”minor axis length”*, and *”energy”*. The most consistent negative contributors were fat-fraction intensity features and heterogeneity-related texture features, including *”gray-level non-uniformity”*, *”size-zone non-uniformity”*, *”dependence entropy”*, and *”IMC”*. This pattern supports the interpretation that greater muscle size and preserved contractile-tissue signal are associated with higher torque, whereas fatty infiltration and increased structural heterogeneity are associated with lower torque. Details of the selected features and global SHAP feature-importance plots are provided in Supplementary Document S.3 (Figure S.2).

## 3 Discussion

Sarcopenia may begin before clear muscle atrophy is visible, yet dedicated imaging approaches for its early detection are not routinely available in clinical practice. Because standard anatomical MRI often includes surrounding muscle, it offers an opportunity for opportunistic assessment of muscle health without additional imaging. In this study, we aimed to identify early sarcopenia-related risk through muscle function, represented here by isometric knee-extension strength. Our results show that conventional measures of muscle quantity, such as CSA, remain informative but do not fully capture muscle functional capacity, particularly in older adults. In contrast, radiomics provided complementary information related to muscle composition, supporting its value as a potential imaging biomarker of early functional decline.

We compared CSA-based and radiomics-based models for predicting isometric knee-extension torque from quadriceps Dixon water-only images and fat-fraction maps across three thigh MRI cohorts acquired at different sites, scanners, and populations. In the healthy cohorts (Dataset-1 + Dataset-2), CSA was numerically the stronger predictor, although the difference did not reach statistical significance. In contrast, once Dataset-3, which included healthy, pre-frail, and sarcopenic older adults, was incorporated, radiomics outperformed CSA, with statistically significant improvement in the full combined cohort under the predefined bootstrap criterion. This pattern was closely related to cohort age and clinical status. As shown in Supplementary Document Figure S.3, when evaluated within individual cohorts, CSA explained the largest share of torque variance in Dataset-2 (*R*^2^ = 0.80), an intermediate share in Dataset-1 (*R*^2^ = 0.56), and the smallest share in Dataset-3 (*R*^2^ = 0.47). In Dataset-1 and Dataset-2, which comprised healthy adults with mean ages of approximately 54–58 years, muscle size remained a relatively faithful surrogate of force-generating capacity. Accordingly, CSA performed best in the Dataset-1 + Dataset-2 combination (*R*^2^ = 0.58). By contrast, Dataset-3 comprises older adults (mean age ≈73 years), including individuals with pre-frailty and sarcopenia, in whom muscle strength often declines faster than muscle mass, consistent with the European Working Group on Sarcopenia in Older People 2 (EWGSOP2) emphasis on low muscle strength as the primary indicator of sarcopenia rather than low muscle mass alone [3]. CSA performance degraded whenever Dataset-3 entered the pool (Dataset-1 + Dataset-3: *R*^2^ = 0.24; full cohort: *R*^2^ = 0.42). These findings indicate that while CSA is a strong predictor of muscle strength in all groups, radiomics captures complementary information related to muscle composition that becomes increasingly important in older adults.

The performance of radiomics might also depended on the type of fat-derived image used. A key methodological distinction is that Dataset-1 used a 2-echo Dixon-derived fat fraction, whereas Dataset-2 and Dataset-3 used multi-echo chemical-shift-encoded proton density fat fraction (PDFF) maps. Unlike a simple 2-point fat signal ratio, PDFF provides a more quantitative and reproducible estimate of muscle fat by accounting for signal decay, multi-peak fat spectrum, and bias related to *T*_1_ weighting and noise. Because radiomic features are calculated directly from voxel intensities, this distinction matters: bias or instability in the underlying map can be interpreted as image texture. This likely contributed to the pattern observed across datasets, where radiomics performed best when both cohorts contributed PDFF maps (Dataset-2 + Dataset-3, *R*^2^ = 0.66–0.67) and less strongly when Dataset-1 was included (*R*^2^ = 0.57–0.59).

The same distinction helps explain why fat fraction-based radiomics outperformed water-only radiomics in every dataset combination. Water-only images are reconstructed after separation of the fat signal and therefore reflect the lean muscle tissue. Moreover, even after intensity normalization and resampling to a common voxel spacing prior to feature extraction, their voxel values remain in arbitrary units and may retain acquisitiondependent differences in relaxation weighting, receive-field inhomogeneity, and effective SNR that global normalization cannot eliminate. By contrast, fat-fraction maps express the relative proportion of water and fat protons within each voxel. Because this ratio is calculated directly from the underlying signal model, common-mode variations—such as coil sensitivity profiles and receiver gain—are mathematically canceled. Fat-fraction maps are therefore intrinsically normalized, representing both lean tissue volume and lipid accumulation on a standardized, physically interpretable scale. This combination is biologically relevant, because strength in aging and early sarcopenia depends not only on the amount of contractile tissue but also on the extent and spatial pattern of fat infiltration. Consistent with this, fat-fraction radiomics was the best or near-best model in all combinations and was also the most stable, varying only between *R*^2^ = 0.57 and 0.66, compared with 0.48–0.58 for water-only radiomics and 0.24–0.58 for CSA. Taken together, these observations support quantitative multi-echo PDFF as the preferred input for composition-based radiomic models intended for multi-site or longitudinal use. The observation that combining water+fat fraction radiomics features did not outperform fat fraction radiomics alone is also biologically and statistically plausible. Because the separated water-only image and the fat fraction images were derived from the same underlying acquisition and were both related to the tissue water-fat composition, a substantial degree of shared information was expected between the two feature sets. As a result, concatenating both modalities may introduce redundancy and collinearity among radiomic predictors rather than adding complementary signal. In this setting, the fat fraction-derived features alone may already capture the dominant compositional variation relevant to muscle strength, such that adding water-only features provides limited incremental benefit and may even complicate model optimization. This interpretation is consistent with the finding that fat fraction-only radiomics provided the most consistently strong performance across the multi-cohort analyses.

The selected radiomic features further support the biological plausibility of the models. Across dataset combinations, SHAP analyses indicated that the selected features fell into two broad groups with consistent directional behavior: size- and signal-magnitude descriptors were positively associated with predicted strength, whereas heterogeneity-related descriptors were negatively associated with it. Features such as *maximum 2D diameter slice, minor axis length*, and water-only *first-order energy* showed positive contributions, indicating that the radiomics pipeline recovered the expected size–force relationship even without explicitly using CSA as an input. In contrast, features such as *gray-level non-uniformity, size-zone non-uniformity, dependence entropy, inverse variance*, and *IMC* showed negative contributions, consistent with greater local disorder and compositional heterogeneity being associated with lower strength. These patterns suggest that radiomics captures aspects of muscle quality that are not represented by a single whole-muscle summary metric such as mean fat fraction (Supplementary Document Figure S.4), which helps explain the limited complementary value of mean fat fraction when added to CSA (Supplementary Document S.4).

Notably, many of the most informative heterogeneity features were extracted not from the original images but from filtered images, including wavelet-, logarithm-, squareroot-, and LBP-transformed images. Conceptually, these filters de-emphasize global intensity level and instead highlight local spatial variation and intensity transitions at different scales. Their repeated selection suggests that the imaging information most relevant to strength lies in fine-grained intramuscular variation rather than in average intensity alone. This is consistent with the limited additional value of mean fat fraction when combined with CSA and further supports the interpretation that local tissue organization contributes meaningfully to muscle function. Because many radiomic features encode overlapping properties, we interpreted these results at the level of stable feature groups (size- and heterogeneity-related) rather than any single selected feature. Although demographic factors such as age, sex, and BMI can influence muscle structure and fat infiltration, we did not explicitly model their effects in this study. Recent literature has already described how these variables relate to muscle volume and intramuscular fat [9]. Instead, our objective was to determine whether radiomics features provide improved prediction of muscle strength compared with conventional imaging measures such as CSA alone or CSA combined with fat fraction. This question is particularly relevant in older adults, given the EWGSOP2 framework, which prioritizes reduced muscle strength over low muscle mass alone in the identification of sarcopenia [3]. Our findings extend existing literature by showing that radiomic features extracted from quadriceps water-only and fat-fraction MRI contain information related to kneeextension strength beyond conventional measures such as CSA and mean fat fraction. Together, these findings support MRI radiomics of the quadriceps as a promising approach for capturing muscle characteristics relevant to strength beyond muscle size alone and have potential to support opportunistic assessment of muscle health and future sarcopenia risk stratification.

This study has some limitations. First, the data was collected across multiple sites and scanner vendors, which is an important strength in terms of real-world representativeness and external validity, but introduced variability in acquisition and reconstruction protocols. While such heterogeneity enhances the robustness of the evaluation, applying harmonization or domain-adaptation methods could stabilize radiomic features across scanners and pipelines, thereby further improving generalizability. ComBat harmonization [26] was attempted but did not adequately resolve inter-scanner variability in our datasets, suggesting that more advanced harmonization approaches may be required in future work. Second, manual segmentation masks were used in strength prediction to eliminate potential segmentation errors from automatic methods and to focus on the impact of radiomic features. The primary aim was to assess whether radiomics captures additional information beyond shape-based biomarkers and whether the selected features are meaningful for predicting strength. Third, strength measurements may be affected by protocol-related variability, as differences in dynamometer calibration and testing protocols can introduce systematic measurement bias. This variability may increase label noise and influence model calibration, potentially limiting generalizability across sites. Fourth, the best strength prediction was achieved using Dixon-derived PDFF maps, which are not uniformly acquired in routine clinical musculoskeletal MRI protocols. Therefore, direct clinical translation of the proposed pipeline may currently be limited by sequence availability. Nevertheless, the predictive value of water-only radiomics suggests that the relevant imaging information is not restricted to quantitative fat mapping alone. This raises the possibility that related biomarkers of muscle quality could also be derived from more widely available anatomical T1-weighted MRI sequences, although this was not evaluated in the present study and requires dedicated validation. In addition, the number of subjects with sarcopenia was limited, so Dataset-3 should be interpreted mainly as capturing older age-related muscle changes rather than sarcopenia specifically. Finally, cross-validation was performed within the available cohorts, but external validation using independent multi-center datasets will be essential to confirm the transferability and robustness of both CSA- and radiomics-based models.

## 4 Methods

### 4.1 Participants

The study was conducted in accordance with the principles of the Declaration of Helsinki and approved by the Institutional Review Board of the National Institute of Environmental Health Sciences, Stanford University, and NYU Langone Health. Written informed consent was obtained from all participants prior to enrollment and study procedures. This study analyzed three MRI cohorts comprising a total of 141 adult participants. Dataset-1 from GESTALT study included 82 healthy adults (36 females, age 22–94 years; Philips MRI) [27]. Dataset-2 consisted of 24 healthy adults (12 females, age 30–79 years; GE MRI) [12, 28]. Dataset-3 included 35 older adults (18 females, age 70–91 years; Siemens MRI), spanning 20 healthy, 13 pre-frail, and 2 subjects with sarcopenia. Pre-frailty was classified using the Fried Frailty Phenotype [29], and sarcopenia was determined according to the EWGSOP2 [3]. Details of the study cohort are presented in Table 2. Representative Dixon water-only and fat-fraction images from healthy subjects across the three cohorts are shown in Supplementary Document Figure S.1, and the full inclusion and exclusion criteria are provided in Supplementary Document S.1.

### 4.2 MRI Acquisition

This study used water-only and fat fraction Dixon MR images, with imaging parameters summarized in Table 2. Dataset-1 was acquired on a Philips Achieva 3T X-series system using a 32-channel cardiac coil, comprising 16 anterior and 16 posterior elements. The Dixon sequence was collected in a single stack, in the central-to-distal thigh region (flip angle 6°; scan time ≈1 min 32 s). Portions of this cohort have been previously described by Cameron et al. [27]. Dataset-2 was acquired on a 3T Signa Premier scanner (GE) using a 16-element anterior array and a 12-element posterior array, with the Dixon sequence collected in two manually overlapped stacks (flip angle 5°; scan time ≈2 min 57 s per stack), as reported in Mazzoli et al. [12, 28]. Dataset-3 was obtained on a 3T Siemens Prisma system using a 16-element anterior array and a 12-element posterior array, with the Dixon sequence likewise acquired in two stacks, with the overlap adjusted to ensure complete thigh coverage (flip angle 5°; scan time ≈1 min 44 s per stack).

### 4.3 MRI Data Analysis

Thigh muscle regions of interest in Dataset-1 were annotated on Dixon images for the left rectus femoris (RF), vastus intermedius (VI), vastus medialis (VM), and vastus lateralis (VL). For Dataset-2 and Dataset-3, the left and right quadriceps muscles (RF, VI, VM, VL) were manually segmented. CSA was calculated as the sum of the maximum CSA of the individual quadriceps muscles.

#### 4.3.1 Skeletal Muscle Strength

Across all datasets, isometric knee-extension torque was assessed using an isokinetic dynamometer, with the HumacNorm system used for Dataset-2 and the Biodex Multi-Joint System PRO (Advantage Software v4X, Biodex Medical Systems, Inc.) used for Dataset-1 and Dataset-3. Five maximum voluntary isometric contractions were performed, and the peak knee extension torque value across the five repetitions was selected. For all measurements, subjects were permitted several submaximal practice efforts prior to the data collection at maximum effort.

### 4.4 Strength Prediction Model

A 5-fold subject-level cross-validation (CV) scheme stratified by age, gender, and strength was used for strength prediction. Quadriceps CSA was computed from the manual muscle segmentations and was used as a baseline predictor in a linear mixed-effects regression model. In parallel, radiomics features were extracted from the four quadriceps muscles—VL, VM, RF, and VI—using PyRadiomics [30]. To ensure voxel-level consistency across cohorts, all images were resampled to the voxel spacing of Dataset-2 prior to feature extraction. Features were computed from both the original images and all PyRadiomics-derived image transformations (e.g., wavelet, gradient, logarithmic, exponential), following established multi-transform radiomic pipelines [31, 32]. For radiomics-based strength prediction, redundant features were first removed by discarding all pairs with correlation coefficients greater than 0.90. The remaining features were then filtered using a score-based threshold derived from held-out validation performance. Next, the retained features were ranked within the training data according to their XGBoost gain, and the ten highest-ranking radiomic features were selected for training an XGBoost regressor [33] to predict continuous knee-extension torque. Hyperparameter optimization for XGBoost was performed using Optuna [34]. The complete feature-selection and modeling pipeline was applied independently to dataset combinations (Dataset-1 + Dataset-2; Dataset-1 + Dataset-3; Dataset-2 + Dataset-3: and Dataset-1 + Dataset-2 + Dataset-3), allowing performance to be evaluated across different cohort configurations. To evaluate agreement between predicted and reference strength values, residual and Bland–Altman analysis [35] was conducted for both the CSA-only and radiomics-based models, estimating mean bias and limits of agreement (LOA) within each dataset combination. Additional methodological details are provided in Supplementary Document S.2, and the overall workflow is summarized in Figure 2.

## Supplementary Material

This is a list of supplementary files associated with this preprint. Click to download.

- CigdemSRSupplementaryDocument.pdf

## Figures and Tables

**Fig. 1:**
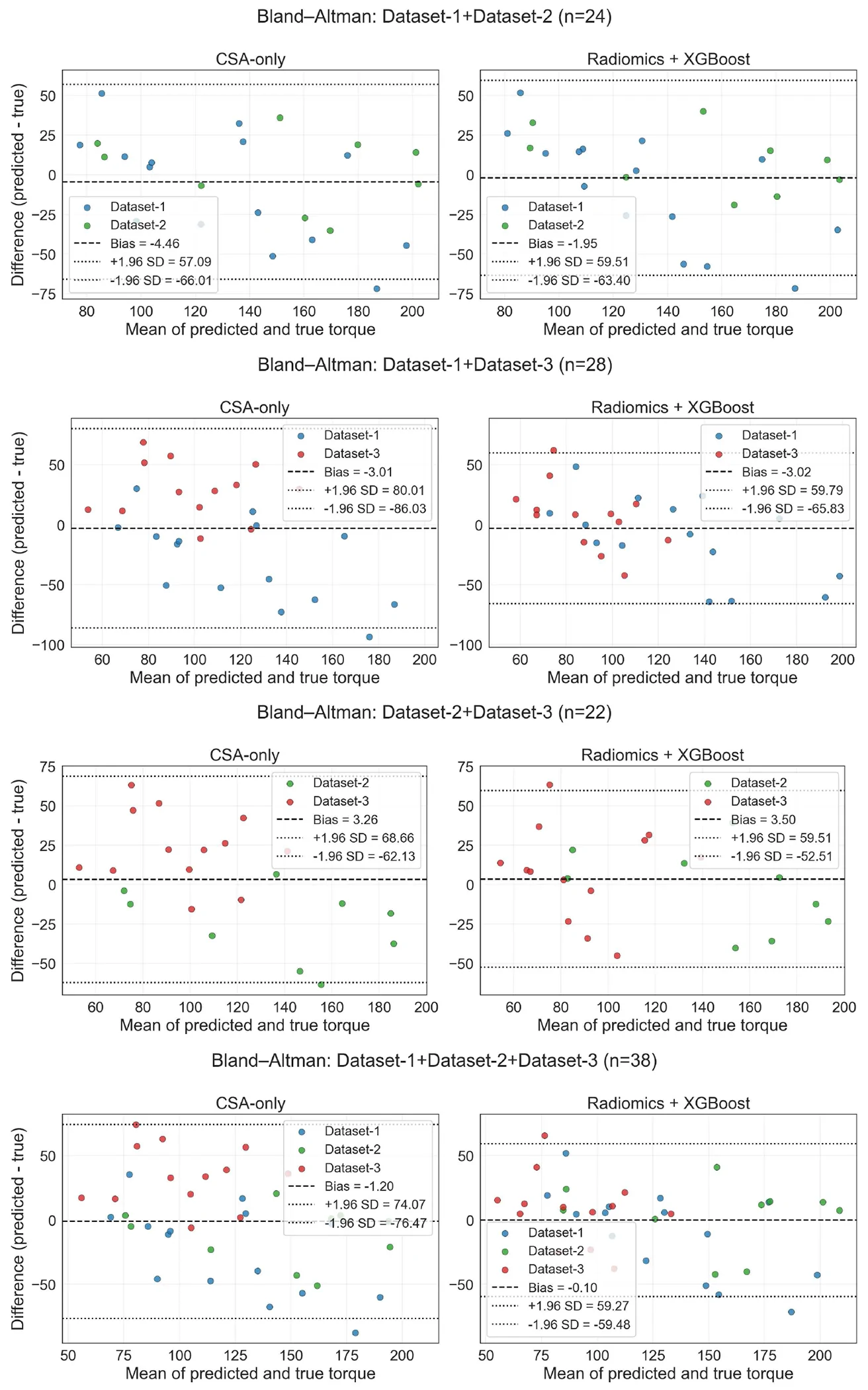
Bland–Altman plots comparing strength predictions from a CSA-only model (linear mixed model) and a radiomics-based XGBoost model across different dataset combinations of fat fraction maps.

**Fig. 2:**
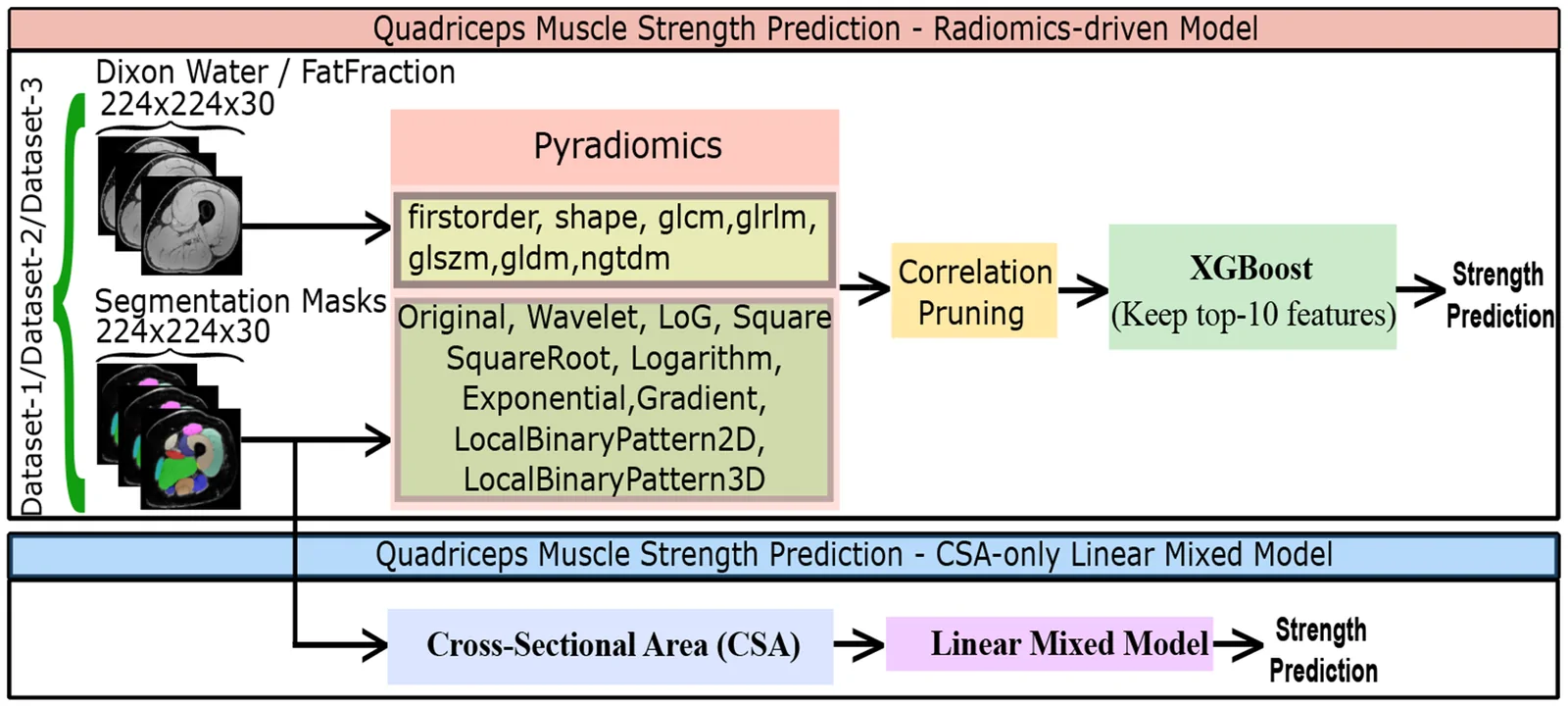
Quadriceps muscle strength prediction using radiomics-driven and CSA-based models. The upper panel illustrates the radiomics-driven pipeline, in which radiomic features are first extracted separately from Dixon water-only images and fat fraction images using the corresponding segmentation masks. These feature sets are then used either individually or in combination (water+fat fraction) across different dataset combinations (Dataset-1, Dataset-2, and Dataset-3) for feature selection and strength prediction using XGBoost. The lower panel shows the CSA-only approach, in which the sum of the maximum cross-sectional areas of the individual quadriceps muscles is used as input to a linear model for strength prediction.

**Table 1:** Comparison of strength prediction performance using maximum cross-sectional area (CSA) derived from MRI masks and radiomics features extracted from different image inputs, including water-only images (Water), fat fraction images (FF), and their combination (Water+FF), using 5-fold cross-validation. RMSE, MAE, and *R*^2^ are point estimates on the held-out test set. For each radiomics model, Δ*R*^2^, ΔRMSE, and ΔMAE represent pairwise subject-level bootstrap comparisons relative to the CSA baseline within the same dataset (Data-) combination, with positive values indicating an increase in *R*^2^ and decreases in RMSE and MAE for the radiomics model. Statistically significant improvements are shown in bold.

Datasets	Image	Method	RMSE	MAE	*R* ^2^	ΔRMSE [95% CI]	ΔMAE [95% CI]	Δ*R*^2^ [95% CI]
Data-1+Data-2	–	CSA	31.1	26.2	0.58	–	–	–
	Water	Radiomics	34.7	27.8	0.48	−3.6 [−6.89, −0.30]	−1.7 [−5.75, 2.49]	−0.103 [−0.231, −0.009]
	FF	Radiomics	30.8	24.5	0.59	0.31 [−3.13, 4.05]	1.70 [−2.12, 5.72]	0.008 [−0.095, 0.111]
	Water+FF	Radiomics	33.8	26.8	0.51	−2.69 [−6.17, 1.30]	−0.57 [−4.60, 3.33]	−0.075 [−0.203, 0.036]
Data-1+Data-3	–	CSA	41.7	33.4	0.24	–	–	–
	Water	Radiomics	32.1	25.0	0.55	**9.6 [3.51, 14.92]**	**8.4 [1.42, 15.12]**	**0.308 [0.119, 0.578]**
	FF	Radiomics	31.6	24.8	0.57	**10.09 [3.59, 16.58]**	**8.62 [1.20, 16.31]**	**0.322 [0.131, 0.633]**
	Water+FF	Radiomics	31.0	23.2	0.58	**10.67 [3.99, 16.37]**	**10.28 [3.09, 16.93]**	**0.338 [0.146, 0.645]**
Data-2+Data-3	–	CSA	32.8	26.9	0.54	–	–	–
	Water	Radiomics	31.2	24.3	0.58	1.6 [−7.25, 11.77]	2.6 [−6.57, 11.78]	0.044 [−0.277, 0.321]
	FF	Radiomics	28.1	23.3	0.66	4.62 [−3.14, 11.63]	3.59 [−4.67, 10.94]	0.121 [−0.099, 0.336]
	Water+FF	Radiomics	27.9	23.6	0.67	4.90 [−3.64, 13.09]	3.36 [−4.67, 11.25]	0.128 [−0.120, 0.356]
Data-1+Data-2+Data-3	–	CSA	37.9	29.5	0.42	–	–	–
	Water	Radiomics	33.6	25.7	0.55	**5.3 [0.82, 10.06]**	5.2 [−0.52, 10.82]	**0.155 [0.024, 0.359]**
	FF	Radiomics	29.9	23.4	0.64	**8.02 [3.06, 12.55]**	**6.06 [0.17, 11.88]**	**0.218 [0.085, 0.399]**
	Water+FF	Radiomics	33.0	26.0	0.57	**5.97 [1.43, 10.48]**	4.98 [−0.45, 10.52]	**0.172 [0.039, 0.352]**

**Table 2:** Cohort demographics and MRI acquisition parameters for thigh imaging. Acq.: acquired, Rec.: reconstructed, std: standard deviation, Δ: time difference between echoes. n: numbers.

	Dataset-1 (Philips)	Dataset-2 (GE)	Dataset-3 (Siemens)		

Participants	Healthy	Healthy	Healthy	Pre-frailty	Sarcopenia
Sex					
Male subjects (n)	46	12	10	6	1
Female subjects (n)	36	12	10	7	1

Age (years)					
Male, mean ± std	56.4 ± 21.9	57.5 ± 15.7	73.2 ± 2.0	74.0 ± 4.1	91
Female, mean ± std	54.0 ± 18.6	58.1 ± 15.6	73.1 ± 3.9	75.6 ± 4.8	72
Age range	22–89 / 25–81	30–78 / 31–77	70–75 / 69–82	70–79 / 72–85	–

Laterality	Left	Bilateral	Bilateral		

Scanner	3T Achieva	3T Signa Premier	3T Prisma		

TR/TE–ΔTE (*ms*)	5.8/1.4–1.2	13.3/1.14–1.14	19.2/1.2–1.49		

Voxel size (Acq.) (*mm*^3^)	1.14×1.14×6	1.76×1.76×6	1.76×1.76×6		

Voxel size (Rec.) (*mm*^3^)	1.14×1.14×6	1.76×1.76×6	0.88×0.88×5		

Matrix (per stack; n)	224×224×30 (1)	256×256×42 (2)	512×352×50 (2)		

Dixon method					
Acquired	2 echoes	12 echoes	12 echoes		
Reconstruction Type	2 echoes	3 echoes	3 echoes		

## Data Availability

The datasets analyzed during the current study are available upon reasonable request.
